# No associations between environmental exposures and stroke severity in a low pollution area in Sweden

**DOI:** 10.1038/s41598-025-06639-w

**Published:** 2025-06-20

**Authors:** Tamar Abzhandadze, Peter Molnar, Adam Viktorisson, Mikael Ögren, Katharina S. Sunnerhagen, Leo Stockfelt, Annie Palstam

**Affiliations:** 1 Institute of Neuroscience and Physiology, The Sahlgrenska Academy, University of Gothenburg, Gothenburg, Sweden; 2 Department of Occupational Therapy and Physiotherapy, Sahlgrenska University Hospital, Gothenburg, Sweden; 3 Occupational and Environmental Medicine, School of Public Health and Community Medicine, Institute of Medicine, Sahlgrenska Academy, University of Gothenburg, Gothenburg, Sweden; 4 Dep of Rehabilitation Medicine, Sahlgrenska University Hospital, Gothenburg, Sweden; 5 Department of Occupational and Environmental Medicine, Sahlgrenska University Hospital, Region Västra Götaland, Gothenburg, Sweden; 6 School of Health and Welfare, Dalarna University, Falun, Sweden

**Keywords:** Air pollution, Noise, Cerebrovascular stroke, Ischemic stroke, Epidemiology, Hemorrhagic stroke, Risk factors, Planetary health, Risk factors, Epidemiology

## Abstract

Mounting evidence supports associations between air pollution and noise exposure and cardiovascular events; however, the relationships at low exposure levels and for stroke outcomes remain uncertain. The aim was to investigate the associations between environmental exposures over 1-year and 10-year periods and both stroke severity and stroke type in a registry-based cohort including people with stroke residing in a low-pollution area of Sweden. Patients with stroke admitted to the Sahlgrenska University Hospital from 2014 to 2019 were included. Stroke severity was assessed with the National Institutes of Health Stroke Scale and stroke types were ischemic and hemorrhagic. Annual residential environmental exposures (road traffic noise (L_Aeq_,_24h_), inhalable particulate matter (PM_10_), and nitrogen oxides (NO_x_)) were assigned from high-resolution dispersion models to participants one year and for ten years prior to stroke, respectively. Of 4066 patients, 1965 (48.3%) were women. The mean (± SD) age was 73.6 (14.0) years. A total of 1563 (28%) had moderate to severe stroke, and 3603 (88.6%) had ischemic stroke. We did not find significant associations between environmental exposures (L_Aeq,24h_, NO_x_, PM_10_) and stroke severity nor stroke type. The generally low levels of exposure and low variance of these environmental factors might explain the lack of observed associations.

## Introduction

Globally, one in four people are affected by stroke during their lifetime. This makes stroke the most common cause of long-term disability among adults with 18 million years lived with disability^1^. In Sweden, about 21 000 people are diagnosed with stroke yearly^2^. Together with World Health Organization (WHO), the European Stroke Organization (ESO) has launched an Action Plan with specific goals for stroke, to be attained by 2030^3^. One goal is to reduce the number of strokes in Europe by 10%, which is anticipated to be attained by targeting lifestyle risk factors such as physical inactivity and by reducing environmental risk factors such as air pollution^3^. However, air pollution is not yet recognized as one of the major risk factors, although evidence is building concerning mechanisms and pathways for the negative effects of ambient particulate matter air pollution on vascular health^4–7^.

Ambient particulate matter (PM) air pollution is the major environmental cause of disease, contributing four million deaths per year, being the reason behind 17% of ischemic heart disease cases and 14% of stroke cases, and causing 24% of deaths in stroke worldwide^8,9^. Further, evidence on associations between air pollution and stroke incidence are building^10,11^. Short-term exposure to air pollution i.e. hours to days of exposure could act as a trigger for stroke and has been associated with incidence of stroke as well as stroke mortality^12,13^, associations being strongest for ischemic stroke type^10,12^. In line with this, long-term exposure to air pollution i.e. months to years, has also been associated with stroke incidence^14,15^. Positive associations between road traffic noise and stroke incidence have also been reported^16–18^.

Inhaled air pollutants initiate events that can lead to stroke, primarily through inducing systemic inflammation, autonomic nervous system dysfunction, and direct neurotoxicity^10^. The inflammatory pathway, triggered by inhaled particulates, releases pro-inflammatory markers and reactive oxygen species that can lead to coagulation, vascular dysfunction, and hypertension. Simultaneously, air pollutants may disrupt autonomic nervous system homeostasis, leading to increased sympathetic tone, vasoconstriction, elevated blood pressure, and cardiac arrhythmias^10^. Ultrafine particles can even breach the blood–brain barrier, potentially accumulating in areas of vascular inflammation and causing direct neurotoxicity and white matter injury. Chronic exposure to these pollutants exacerbates subclinical manifestations such as atherosclerosis, endothelial dysfunction, hypertension, and cardiac arrhythmias, further increasing the risk of stroke^10^. The mechanisms leading to ischemic and haemorrhagic strokes differ in their emphasis on these pathways. Ischemic strokes are more closely linked to atherosclerosis and cardio-embolism, where pollutants contribute to plaque instability and rupture, leading to vessel occlusion. Cardiac arrhythmias, particularly atrial fibrillation, induced by air pollution can also increase the risk of embolic strokes^10^. In contrast, haemorrhagic strokes are more associated with acute vasoconstriction and changes in vascular resistance resulting from autonomic nervous system dysfunction. Furthermore, chronic exposure to air pollution may lead to microvascular damage and cerebral microbleeds, predisposing individuals to haemorrhagic events^10^.

Beyond incidence of stroke, associations between air pollution and stroke severity have been investigated, showing inconclusive results. In Barcelona, a study of more than 2700 patients with stroke found that traffic noise was associated with a more severe stroke in the acute phase, but no association between PM_2.5_ and initial stroke severity^19^. In line with this finding, a study of approximately 2500 patients with stroke in London found no association between PM_10_ or NO_2_ and initial stroke severity^20^. Another study, including more than 3000 patients with ischemic stroke in the city of Corpus Christi, Texas, found that PM_2.5_ was associated with stroke severity, but only in socially disadvantaged areas^21^.

Air pollution levels differ geographically, being higher in Asian countries than in Europe and North America^22^, also reflected in findings of stronger association between air pollution and stroke in more polluted regions in the world^10,13^. In Sweden, air pollution levels are generally low, and have improved even further during the recent years^23^. In a Swedish study on traffic related air pollution and stroke incidence with longitudinal data, no clear associations were found, possibly reflecting relatively low levels of pollutants^24^. In another Swedish study with low levels of air pollution, associations with stroke incidence were only found for black carbon, but neither for PM_10_ or PM_2.5_^25^. In yet another Swedish study, associations were found between long-term exposure to NO_x_, risk factors for stroke, and stroke incidence^26^.

There is thus evidence of associations between both air pollution and noise exposure and cardiovascular events, including stroke. However, the relationships at low exposure levels and for admission stroke severity or type of stroke remain uncertain. Therefore, this study aims to analyse possible associations between environmental exposures the year before stroke on admission stroke severity.

Also, to investigate possible associations between environmental exposures over the decade preceding hospital admission on the severity of stroke and stroke type.

## Methods

We adhered to the guidelines outlined by the Strengthening the Reporting of Observational Studies in Epidemiology (STROBE) for reporting of this study.

### Study design and study sample

This registry-based cohort study utilized data compiled from multiple registries and sources. Väststroke, Gothenburg’s local stroke registry, records information on patients admitted to stroke units at Sahlgrenska University Hospital (SU), which operates three stroke units. One of these units provides thrombolysis services for Gothenburg’s urban and rural populations, and thrombectomy services for the Västra Götaland County.

Data on stroke outcomes were obtained from Riksstroke, Sweden’s national quality registry for stroke care^27^. We collected data on stroke patients admitted to SU’s stroke units between 2014 and 2019. Environmental and climate data were modelled by the Swedish Meteorological and Hydrological Institute (SMHI). Additionally, socio-economic data were retrieved from Statistics Sweden’s Longitudinal Integrated Database for Health Insurance and Labour Market Studies (LISA), which contains comprehensive information on Swedish citizens^28^. Patients were included if they were 18 years or older at the time of the stroke, had a clinically confirmed index stroke, and a diagnosis consistent with the International Classification of Diseases, 10th Revision (ICD-10), specifically codes I61 (hemorrhagic stroke) and I63 (Ischemic Stroke). Furthermore, information on stroke severity upon hospital admission was necessary for inclusion. We excluded patients with incomplete data on environmental exposure variables, and a history of previous stroke (Table 1).

**Table 1:** Characteristics of the study sample (n = 4066) and unadjusted associations between covariates and moderate/severe stroke.

Characteristics	Mild stroke NIHSS 0–5 p (n = 2503)*	Moderate/severe stroke NIHSS 6–36 p (n = 1563)*	Crude associations Odds ratio (95% CI)^#^
Age, years			1.03 (1.02–1.03)
Mean (± SD)	71.7 (14.1)	76.6 (13.3)	
Median (IQR)	74 (18)	79 (18)	
Sex, men	1395 (55.7)	706 (45.2)	0.65 (0.58–0.74)
Education^1^			
University education (≥ 13 years)	658 (28.4)	288 (19.0)	Ref
Secondary school (10–12 years)	969 (40.1)	610 (40.3)	1.50 (1.27–1.78)
Primary school (≤ 9 years)	773 (31.5)	614 (40.6)	1.92 (1.61–2.28)
Own home with/without community cervices^2^	2391 (95.6)	1344 (86.7)	0.40 (0.36–0.47)
Living alone, yes^3^	1150 (46.4)	786 (51.0)	1.21 (1.06–1.37)
Need of help before stroke, yes^4^	2016 (84.8)	1026 (71.9)	2.18 (1.86–2.56)
Birth country, Sweden	2031 (81.1)	1260 (80.6)	Ref
Other	472 (18.9)	303 (19.4)	1.04 (0.88–1.21)
Income in 1000 SEK (range)^5^			
High income (2003.0–94,009.0)	960 (38.6)	422 (27.4)	Ref
Medium income (1471.0–1999.0)	774 (31.1)	531 (34.5)	1.76 (1.50–2.06)
Low income (0.0–1470.0)	756 (30.4)	585 (28.0)	1.56 (1.33–1.83)
Atrial fibrillation, yes^6^	513 (20.5)	597 (38.4)	2.41 (2.10–2.78)
Previous TIA, yes^7^	186 (7.5)	74 (4.8)	0.63 (0.74–0.82)
Diabetes, yes^8^	444 (17.7)	292 (18.7)	1.07 (0.91–1.26)
Smoking, yes^9^	340 (15.1)	160 (12.4)	0.80 (0.65–0.97)
Stroke type			
Haemorrhage (I61)	179 (7.2)	284 (18.2)	Ref
Ischemic stroke (I63)	2324 (92.8)	1279 (81.8)	0.35 (0.28–0.42)
Reperfusion treatment, yes			NR
Thrombolysis^10^	203 (8.8)	403 (31.6)	
Thrombectomy^11^	66 (2.8)	454 (35.6)	
Admission stroke severity (NIHSS)			NR
Mean (± SD)	1.5 (1.6)	14.0 (6.3)	
Median (IQR)	1 (3)	13 (11)	
Length of hospital stay, days			1.04 (1.04–1.05)
Mean (± SD)	11.8 (12.4)	21.7 (20.0)	
Median (IQR)	7 (11)	16 (22)	

*Patient characteristics were presented as the number of patients (No.) and valid column percentages (%), unless otherwise stated*;* Ref., Reference; NIHSS, National Institutes of Health Stroke Scale; CI, confidence interval; SD, standard deviation; IQR, interquartile range; TIA, transient ischemic attack. Variables with missing data (total sample, n [%]): ^1^102 (2.5%), ^2^15 (0.4%), ^3^46 (1.1%), ^4^262 (6.4%), ^5^38 (0.9%), ^6^12 (0.3%), ^7^22 (0.5%), ^8^2 (< 0.01%), ^9^530 (13.0%), ^10^12 (0.3%), ^11^348 (8.6%). Statistics: Unadjusted associations between moderate to severe stroke severity at admission and covariates were analyzed using a binary logistic regression model. NR indicates that data are not relevant for reporting, as reperfusion treatment was provided based on admission NIHSS scores.

### Procedure

Data sources were merged by the registry holders using unique Swedish personal identification numbers, and the final dataset was pseudonymized to protect participant privacy. Multiple registries and datasets were used to capture the diverse range of information needed for the study. Väststroke provided data on stroke severity and type, while Riksstroke contributed information on reperfusion treatments, medications, comorbidities, and living conditions. Geographic Information Systems (GIS) enabled the assignment of yearly mean exposure levels at participants’ residential addresses based on these data sets. LISA contributed data on education and income levels^28^. Healthcare professionals at stroke units recorded the data in Väststroke and Riksstroke, whereas governmental agencies entered the data for SMHI and LISA.

### Variables and definitions

#### Outcomes

The primary outcome of this study was stroke severity at admission, assessed using the National Institute of Health Stroke Scale (NIHSS)^29^. The NIHSS total score ranges from 0 (no stroke symptoms) to 42 (severe stroke with multiple symptoms). If NIHSS data were missing from the registry, one author supplemented the information by reviewing patients’ medical records^30^. In this study, an outcome category moderate/severe stroke was defined as an NIHSS score ≥ 6, while a mild stroke was defined as an NIHSS score ≤ 5^31^.

The secondary outcome was stroke type. Ischemic stroke (ICD-10 code I63) was an outcome category and defined as a stroke caused by a reduction in blood flow to a part of the brain, typically due to an obstruction in the arteries supplying blood to the brain^32^. Intracerebral hemorrhage (ICD-10 code I61) was a reference category and defined as non-traumatic bleeding within the brain tissue, distinct from bleeding in the ventricles or subarachnoid space^32^.

#### Environmental exposures

There were three primary explanatory variables in this study: the mean annual A-weighted 24-h equivalent sound pressure level (L_Aeq,24h_), the mean annual concentrations of nitrogen oxides (NO_x_), and the mean annual concentrations of particulate matter with a diameter of 10 µm or less (PM_10_). Data on all primary explanatory variables were collected for the ten years preceding the stroke. Exposures were averaged across all previous addresses for participants who moved during the study period. For the main analyses, exposure data from one year before the stroke were used. In sensitivity analyses, we calculated the mean exposure levels over the entire 10-year period.

**Fig. 1:**
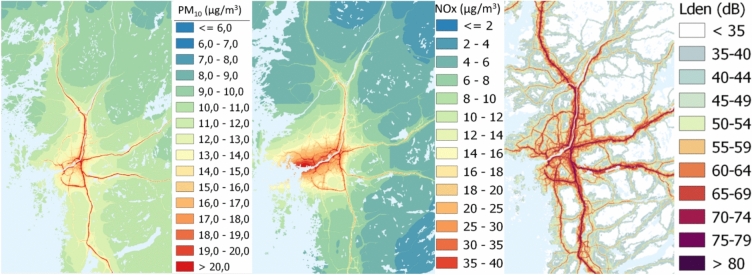
Maps of the greater Gothenburg area, Sweden, with the levels of PM_10_ (µg/m^3^), NOx (µg/m^3^) and road traffic noise (dB) for the model year 2018, retrieved from Molnár and Ögren^33,34^. Air pollution and noise maps for SCAPIS environment (Version 1) [Data set]. University of Gothenburg. The maps were created in QGIS 3.22 (https://qgis.org/) by importing the geo-tiff files created using high resolution Gaussian dispersion models as described elsewhere 10.5878/btxv-v698^23,35^. The conversion for Lden is: Lden = LAEq,24h + 3.1.

Concentrations of PM_10_ and nitrogen oxides (NO_x_) were modeled at a dynamic resolution of up to 50 × 50 m^2^, using methods detailed elsewhere^23^. In brief, emission inventories were compiled for the years 2000, 2011, and 2018, incorporating data on both local sources and long-range transport. Concentrations for intermediary years were interpolated and adjusted for meteorological variations. Local emissions were modeled using dispersion models, while long-range transport emissions were estimated with a bias-corrected chemical transport model. The modeled particle levels were validated by comparing them with data from quality-controlled monitoring stations. Illustrative maps and Geo files can be accessed through the Swedish National Data Service^33,34^. Illustrative maps of mean NO_x_ and mean PM_10_ concentrations, as well as mean levels of L_Aeq,24h_ in the study area for the year 2018 are presented in Fig. 1.

Residential exposure to road traffic noise levels was based on traffic intensity data for the years 2000, 2011, and 2018, modeled using previously described techniques^35^. In brief, the 24-h A-weighted equivalent sound pressure level (L_Aeq,24h_) from road traffic was estimated at the most exposed façade of each residence, using a dynamic grid with resolutions up to 25 m.

Yearly average exposure levels were assigned to all study participants using QGIS software (version 3.22, QGIS Development Team), based on their individual address history obtained from Statistics Sweden. The addresses were automatically geocoded and then manually checked and corrected for accuracy.

#### Variables and covariates

The following covariates were analyzed: sex (male or female) and age (as a continuous variable). Income was based on disposable income during the year before the stroke, expressed in thousands of Swedish Krona (SEK) (average exchange rates for 2020 provided by the Swedish Riksbank (10.5 SEK = 1 EUR, 11.8 SEK = 1 GBP). Education was categorized into three levels: primary school (≤ 9 years), secondary school (10–12 years), and university education (≥ 13 years). Living circumstances were classified as either living alone or living with someone else. The need for assistance was defined as requiring help with daily activities or not. Accommodation was categorized as living in one’s own home, with or without community assistance, or residing elsewhere, including nursing homes. Reperfusion treatment was defined as either thrombolysis or thrombectomy. The length of hospital stay was measured as the number of days from admission to discharge. Additionally, data from Riksstroke were analyzed to include the following risk factors and comorbidities as covariates in multivariable regression models: diabetes, atrial fibrillation, and transient ischemic attack (TIA). For descriptive statistics, we included data on the use of thrombolysis and thrombectomy, as well as the length of hospital stay (in days).

### Data analyses

Differences between included and excluded patients were assessed using the Chi-squared test for sex and stroke type, and the Mann–Whitney U test for age and stroke severity. Descriptive statistics for the sample were presented as frequencies with valid percentages (%), means with standard deviations (SD), and medians with interquartile ranges (IQR).

Crude associations between admission stroke severity groups and covariates were examined using binary logistic regression, with results reported as odds ratios (OR) and 95% confidence intervals (95% CI). Group differences in the primary independent variables, admission stroke severity categories, and stroke types were analyzed using independent sample t-tests.

The primary outcome variable was stroke severity at admission, defined as moderate to severe stroke (NIHSS ≥ 6) and the reference category was a mild stroke (NIHSS ≤ 5)^31^. We performed individual multivariable logistic regression analyses with NO_x_, PM_10_, and L_Aeq,24h_ as the main explanatory variables. The models were adjusted for covariates, including sex, age, education, country of birth, individual income in the year before the stroke, atrial fibrillation, previous transient ischemic attack, diabetes, accommodation type, and cohabitation status. Smoking was not included in the multivariable models due to the high proportion of missing values (13%). These variables were chosen based on authors’ clinical reasoning and previous studies^36–38^. Results were presented as OR with 95% CI. A similar regression analysis approach was applied to the secondary outcome, which was stroke type. In this analysis, hemorrhagic stroke served as the reference category, while ischemic stroke was the studied category. For sensitivity analyses, we calculated the mean levels of NO_x_, PM_10_, and L_Aeq,24h_ over the 10 years preceding the index stroke.

### Ethics approval and consent to participate

Ethical approval has been granted by the Swedish Ethical Review Authority for the project in 2016-05-02 (346-16) with approved amendment 2020-06-15 (2020-02793). Collected data were protected by confidentiality regulations for Public Access to Information. Secrecy Act (2009:400), Chapter 24. The data were handled in accordance with the European Union (EU) General Data Protection Regulation (GDPR) and Swedish law (2018:218), which supplemented the GDPR. The Declaration of Helsinki was not relevant to this project, which was based on data generated in public registries. Informed consent: According to the Swedish Ethical Review Authority, quality registers are exempt to the general rule of patient consent according to the Personal Data Act (Swedish law No. SFS 1998:204).

## Results

**Fig. 2:**
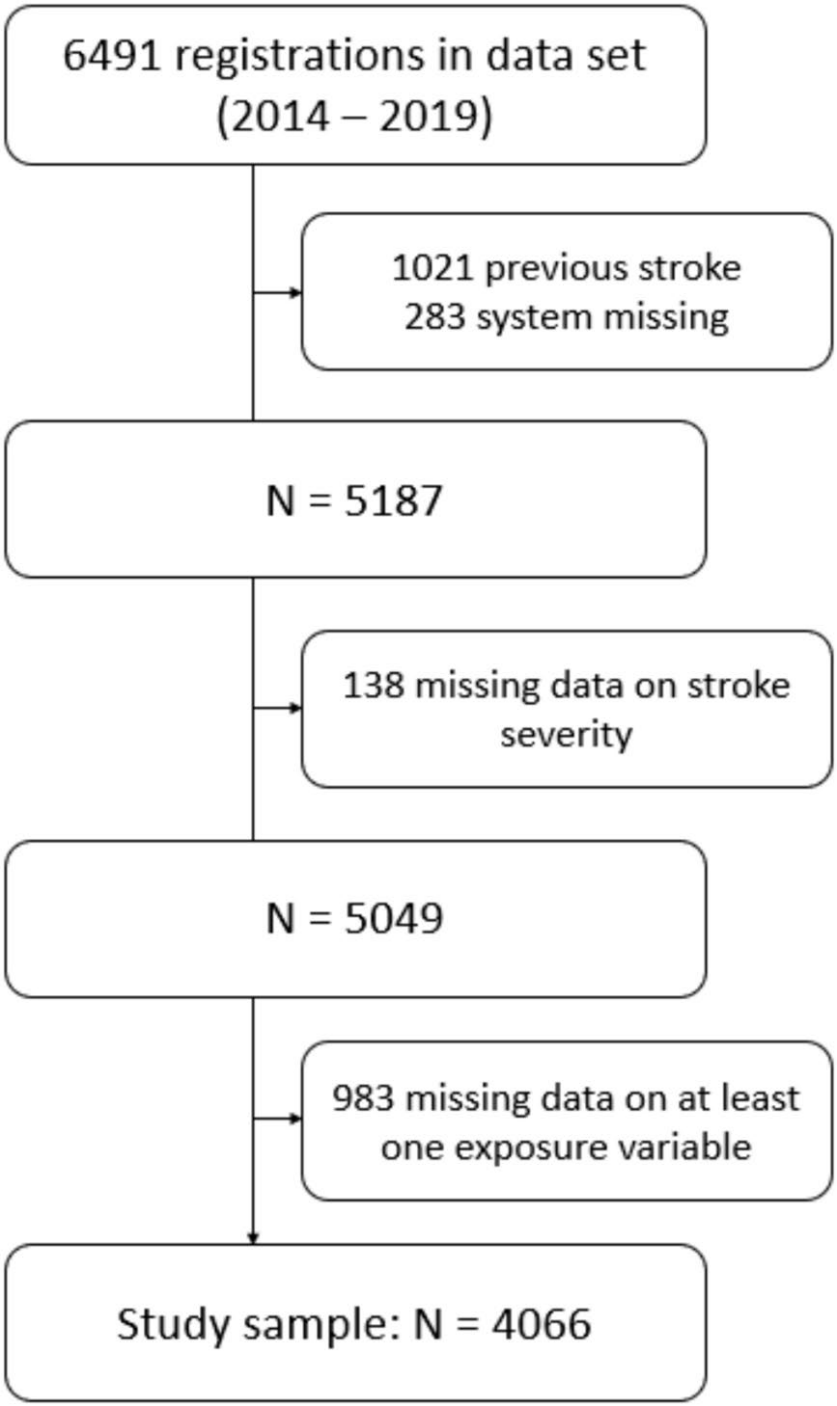
Flowchart illustrating participant inclusion in the study sample.

We included 4066 patients with a first-ever stroke in the study (Fig. 2). There were no significant differences between included (n = 4066) and excluded patients (n = 2425) in terms of sex (p = 0.06), stroke type (p = 0.13), or age (p = 0.13). However, included patients had significantly more severe strokes compared to excluded patients (p < 0.01), with mean (± SD) NIHSS scores of 5.6 (6.7) and 6.3 (7.3), respectively.

### Sociodemographic, stroke-related characteristics, and environmental exposures

Of the 4066 included patients, 1965 (48.3%) were women, and the mean age was 73.6 years (± 14.0). A total of 3603 patients (88.6%) had ischemic strokes. Detailed information on the study sample, stratified by stroke severity, is presented in Table 1.

The average environmental exposures one year before stroke were 55.05 dB for LAeq,24h, 15.30 µg/m3 for NOx, and 14.39 µg/m3 for PM₁₀. The average environmental exposures over the 10-year period prior to stroke were 54.78 dB for LAeq,24h, 16.41 µg/m3 for NOx, and 15.09 µg/m3 for PM₁₀ (Table 2).

**Table 2:** Descriptive statistics of environmental exposures for the year prior to stroke and mean Values over the 10 years before stroke.

	Mean	SD	Median	Maximum	Minimum	Range
1 year before stroke						
L_Aeq,24h_ (dB)	55.05	6.90	55.19	72.89	17.36	55.54
NO_x_ (µg/m^3^)	15.30	7.76	13.75	53.90	2.99	50.91
PM_10_ (µg/m^3^)	14.39	1.93	14.27	29.54	9.30	20.23
Mean of 10 years before stroke						
L_Aeq,24h_ (dB)	54.78	6.69	55.03	72.10	17.21	54.89
NO_x_ (µg/m^3^)	16.41	7.71	15.04	62.60	3.62	58.99
PM_10_ (µg/m^3^)	15.09	1.77	14.93	30.43	10.38	20.05

L_Aeq,24h_: the A-weighted 24-h equivalent sound pressure level in dB; NO_x_: the concentration of nitrogen oxides in µg/m^3^; PM_10_: the concentration of particulate matter with a diameter of < 10 µm in µg/m^3^. SD, standard deviation.

### Environmental exposures and admission stroke severity

There were no significant associations between L_Aeq,24h_ (p = 0.29), NO_x_ (p = 0.88), or PM_10_ (p = 0.08), and admission stroke severity. In multivariable binary logistic regression models, none of the environmental exposure variables significantly explained moderate to severe stroke at admission (Table 3). The complete multivariable models are presented in eTable 1, eTable 2, and eTable 3.

**Table 3:** Results of binary logistic regression analyses examining the associations between moderate to severe stroke at admission and environmental exposures.

Primary independent variables	Mild stroke^#^ (n = 2503)	Moderate to severe^#^ stroke (n = 1563)	Multivariable model^##^	
	Mean (± SD)	Mean (± SD)	Odd ratio (95% CI)	P value
L_Aeq,24h_	55.14 (6.75)	54.90 (7.12)	0.97 (0.93–1.02)	0.28
NO_x_	15.31 (7.77)	15.28 (7.75)	0.98 (0.94–1.03)	0.43
PM_10_	14.43 (1.91)	14.32 (1.97)	0.86 (0.72–1.03)	0.09

^#^A T-test was used for group comparisons. L_Aeq,24h,_ the A-weighted 24-h equivalent sound pressure level. NO_x_, the concentration of nitrogen oxides in µg/m^3^. PM_10_, the concentration of particulate matter with a diameter < 10 µm, in µg/m^3^.

^##^The multivariable binary logistic regression analysis. Odd ratios were calculated with a 5-unit increment for the exposure variables. Regression model was adjusted for sex, age, education, country of birth, individual income the year before the stroke, atrial fibrillation, previous transient ischemic attack, diabetes, accommodation, and cohabitation status. CI indicates confidence intervals. N = 3875, due to missing values.

### Environmental exposures and ischemic stroke

There were no significant associations between L_Aeq,24h_ (p = 0.54), NO_x_ (p = 0.58), or PM_10_ (p = 0.38) and stroke types. The multivariable logistic regression analysis revealed that none of the environmental exposure variables were significant predictors of ischemic stroke (Table 4).The complete multivariable models are provided in eTable 4, eTable 5, and eTable 6.

**Table 4:** Results of binary logistic regression analyses examining the associations between ischemic stroke and environmental exposures.

Primary independent variables	Haemorrhagic stroke^#^ (n = 463)	Ischemic stroke^#^ (n = 3603)	Multivariable model^##^	
	Mean (± SD)	Mean (± SD)	Odd ratio, (95% CI)	P value
L_Aeq,24h_	54.86 (7.17)	55.07 (6.86)	1.02 (0.95–1.09)	0.56
NO_x_	15.11 (7.56)	15.32 (7.79)	1.01 (0.95–1.08)	0.75
PM_10_	14.31 (1.93)	14.40 (1.93)	1.09 (0.85–1.42)	0.49

^#^A T-test was used for group comparisons. L_Aeq,24h_, the A-weighted 24-h equivalent sound pressure level in dB. NO_x_, the concentration of nitrogen oxides in µg/m^3^. PM_10_, the concentration of particulate matter with a diameter < 10 µm, in µg/m^3^.

^##^The multivariable binary logistic regression analysis. Odd ratios were calculated with a 5-unit increment for the exposure variables. Regression model was adjusted for sex, age, education, country of birth, individual income the year before the stroke, atrial fibrillation, previous transient ischemic attack, diabetes, accommodation, and cohabitation status. CI indicates confidence intervals. N = 3875, due to missing values.

### Sensitivity analyses mean of 10 y exposure

In multivariable binary logistic regression analyses, higher PM_10_ levels were significantly associated with marginally lower odds of experiencing a moderate to severe stroke (OR = 0.82; p = 0.04; eTable 7). No significant associations were observed for L_Aeq,24h_, or NO_x_ exposures, eTable 8 and eTable 9, respectively. Additionally, ischemic stroke was not significantly associated with any of the environmental exposure variables (eTable 10, eTable 11, eTable 12).

## Discussion

In this registry-based cohort study, no positive associations were found between long-term environmental exposures to air pollution and road traffic noise with initial stroke severity or stroke subtype in 4,066 patients with acute stroke. The levels of particulate matter air pollution and noise were, however, relatively low across all tested exposures and subgroups.

Similar to prior studies, initial stroke severity was not associated with long-term exposure to particulate matter air pollution^19,20^. Further, there were no associations between noise and initial stroke severity, which is contradictory to the previous findings on this topic, showing an association between road traffic noise and initial stroke severity among residents in Barcelona^19^. However, in that study noise levels were somewhat higher, and the associations were significant only regarding the highest quartile of noise levels, which may complicate comparison with the present study. However, in sensitivity analyses an association was found between higher exposure to PM_10_ and less severe stroke. This could possibly be due to multiple statistical testing, or perhaps the low exposure level and limited variability in the PM_10_ variable. However, we could not speculate further on this finding.

One aspect to keep in mind when interpreting our results is that the levels of air pollution were quite low compared with other studies^14,20^. Also, levels of noise were at the lower end compared to studies in the field^17^. The mean levels of PM_10_ in our study corresponded to the WHO 2021 air quality guidelines level of 15 µg/m^3^ as an annual average^22^.

There are several strengths and limitations of this study. An observational design cannot establish causal relationships between environmental exposures and stroke outcomes. The reliance on registry data may also introduce potential biases due to missing or incomplete data, which could affect the accuracy of the analyses. The low levels of exposure and low exposure contrast may have contributed to the lack of significant associations observed in our study. Another aspect that could influence our results is that we were not able to assign PM_2.5_ in our data, but only had access to PM_10_. This is a limitation since the evidence on air pollution affecting stroke incidence is strongest regarding PM_2.5_^4–6,10^. Long-term levels of PM_10_ and PM_2.5_ are however highly correlated (r = 0.85) in this setting^39^. Moreover, the assessment of environmental exposures was based on residential address data and did not account for individual variations in time spent outdoors or occupational exposures. We did, however, follow participants residential history and assign exposure from high-resolution models of good quality. Confounding factors, such as socioeconomic status and pre-existing health conditions, were considered, but residual confounding may still influence the results. Moreover, we did not include smoking in the multivariable models due to the high proportion of missing values, which may influence the results.

As a conclusion, the environmental exposures such as noise levels, nitrogen oxides, and particulate matter did not significantly influence stroke severity or type. Considered that the levels of air pollution were quite low, there are several clinical, and scientific implications of the study. First, these findings suggest that factors beyond environmental exposures should be considered when assessing stroke outcomes. While environmental factors are known to be associated with stroke incidence, this study indicates they may not play a critical role in determining stroke severity or type when hit by stroke in areas with low level of pollution. Second, from a public health perspective, while reducing environmental pollutants is crucial for overall health, these findings suggest that such interventions might not directly reduce the severity of strokes, and perhaps stoke severity is determined by other stronger predictors as age, sex, pre-morbid conditions, etc. However, in return, pre-morbid conditions, such as high blood pressure, is related to high levels of environmental exposures, associations which could be present in our data but not investigated in this study. Third, the results underscore the complexity of stroke aetiology and highlight the need for further research into other potential determinants of stroke outcomes. This could include genetic factors, social determinants of health, or other environmental exposures not covered in this study.

## Supplementary Information

Supplementary Tables.

## Data Availability

The datasets generated and analysed during the current study are not publicly available due to ethical restrictions but are available from the corresponding author on reasonable request.
