# Supplementary material for: No associations between environmental exposures and stroke severity in a low pollution area in Sweden
Source: Sci Rep. 2025 Jun 20;15:20218. doi: 10.1038/s41598-025-06639-w (PMC12181234; doi:10.1038/s41598-025-06639-w)
Supplement: Supplementary file 1 — Supplementary Tables. [file 41598_2025_6639_MOESM1_ESM.pdf]

ONLINE SUPPLEMENT

No associations between environmental exposures and stroke severity in a low pollution area in Sweden

Supplementary Tables

eTable 1. Results of multivariable logistic regression analyses examining the associations between moderate to severe stroke at admission and Equivalent Continuous Sound Level ($L_{Aeq,24h}$) one year before the index stroke.

eTable 2. Results of multivariable logistic regression analyses examining the associations between moderate to severe stroke at admission and the concentration of Nitrogen Oxides (NOx) one year before index stroke.

eTable 3. Results of multivariable logistic regression analyses examining the associations between moderate to severe stroke at admission and the concentration of a particulate matter with a diameter of <10 micrometres (PM_{10}) one year before index stroke.

eTable 4. Results of multivariable logistic regression analyses examining the associations between ischemic stroke and 10 years mean of Equivalent Continuous Sound Level ($L_{Aeq,24h}$).

eTable 5. Results of multivariable logistic regression analyses examining the associations between ischemic stroke and 10 years mean concentration of Nitrogen Oxides (NOx)

eTable 6. Results of multivariable logistic regression analyses examining the associations between ischemic stroke and 10 years mean concentration of a particulate matter with a diameter of <10 micrometres (PM_{10}).

eTable 7. Results of multivariable logistic regression analyses examining the associations between ischemic stroke and Equivalent Continuous Sound Level ($L_{Aeq,24h}$) one year before the index stroke.

eTable 8. Results of multivariable logistic regression analyses examining the associations between ischemic stroke at admission and the concentration of Nitrogen Oxides (NOx) one year before index stroke.

eTable 9. Results of multivariable logistic regression analyses examining the associations between ischemic stroke and the concentration of a particulate matter with a diameter of <10 micrometres (PM_{10}) one year before index stroke.

eTable 10. Results of multivariable logistic regression analyses examining the associations between ischemic stroke and a 10-year mean of the Equivalent Continuous Sound Level ($L_{Aeq,24h}$).

eTable 11. Results of multivariable logistic regression analyses examining the associations between ischemic stroke and a 10-year mean concentration of Nitrogen Oxides (NOx).

eTable 12. Results of multivariable logistic regression analyses examining the associations between ischemic stroke and a 10-year mean concentration of a particulate matter with a diameter of <10 micrometres (PM_{10}).

eTable 1. Results of multivariable logistic regression analyses examining the associations between moderate to severe stroke at admission and Equivalent Continuous Sound Level ($L_{Aeq,24h}$) one year before the index stroke.

Variable	OR	95 % Confidence Interval		P-value
$L_{Aeq,24h}$ (dB)	0.97	0.93	1.02	0.28
Female sex	1.31	1.13	1.52	< 0.001
Age	1.01	1.01	1.02	< 0.001
Atrial fibrillation, yes	2.06	1.77	2.40	< 0.001
University education (≥ 13 years), Ref				
Secondary school (10–12 years)	1.43	1.19	1.72	< 0.001
Primary school (≤9 years)	1.45	1.19	1.76	< 0.001
High income (2003.0 – 94009.0), Ref				
Medium income (1471.0 – 1999.0)	0.99	0.82	1.19	0.92
Low income (0.0 – 1470.0)	1.15	0.96	1.39	0.13
Diabetes, yes	0.94	0.79	1.13	0.56
Birth country, Sweden	0.94	0.78	1.13	0.52
Transient ischemic attack, yes	0.51	0.38	0.68	< 0.001
Living in nursing homes	2.00	1.67	2.42	< 0.001
Living alone, yes	1.28	1.10	1.50	< 0.01

Abbreviations: OR, odds ratio; $L_{Aeq,24h}$ an Equivalent Continuous Sound Level in dB, the year before the stroke. OR is for a 5-unit increment on $L_{Aeq,24h}$. Outcome: moderate to severe stroke defined as ≥ 6 points on the National Institutes of health stroke scale (ref. mild stroke, ≤ 5). Income in 1000 SEK.

Statistics: Binary logistic regression, multivariable model, n = 3875

eTable 2. Results of multivariable logistic regression analyses examining the associations between moderate to severe stroke at admission and the concentration of Nitrogen Oxides (NOx) one year before index stroke.

Variable	OR	95 % Confidence Interval		P-value
NOx (μg/m³)	0.98	0.94	1.03	0.43
Female sex	1.31	1.13	1.52	< 0.001
Age	1.01	1.01	1.02	< 0.001
Atrial fibrillation, yes	2.06	1.77	2.40	< 0.001
University education (≥ 13 years), Ref				
Secondary school (10–12 years)	1.43	1.19	1.72	< 0.001
Primary school (≤9 years)	1.45	1.19	1.76	< 0.001
High income (2003.0 – 94009.0), Ref				
Medium income (1471.0 – 1999.0)	0.99	0.82	1.19	0.94
Low income (0.0 – 1470.0)	1.16	0.96	1.40	0.13
Diabetes, yes	0.95	0.79	1.14	0.58
Birth country, Sweden	0.94	0.79	1.13	0.53
Transient ischemic attack, yes	0.51	0.38	0.69	< 0.001
Living in nursing homes	2.01	1.67	2.41	< 0.001
Living alone, yes	1.28	1.10	1.50	< 0.01

Abbreviations: OR, Odds Ratio; NOx: the concentration of Nitrogen Oxides in μg/m3 the year before the stroke. OR is for a 5-unit increment on NOx. Outcome: moderate to severe stroke defined as ≥ 6 points on the National Institutes of health stroke scale (ref. mild stroke, ≤ 5). Income in 1000 SEK.

Statistics: Binary logistic regression, multivariable model, n = 3875

eTable 3. Results of multivariable logistic regression analyses examining the associations between moderate to severe stroke at admission and the concentration of a particulate matter with a diameter of <10 micrometres (PM_{10}) one year before index stroke.

Variable	OR	95 % Confidence Interval		P-value
PM_{10} (µg/m³)	0.86	0.72	1.02	0.09
Female sex	1.31	1.13	1.51	< 0.001
Age	1.01	1.01	1.02	< 0.001
Atrial fibrillation, yes	2.06	1.76	2.40	< 0.001
University education (≥ 13 years), Ref				
Secondary school (10–12 years)	1.43	1.19	1.72	< 0.001
Primary school (≤9 years)	1.45	1.19	1.76	< 0.001
High income (2003.0 – 94009.0), Ref				
Medium income (1471.0 – 1999.0)	0.99	0.82	1.19	0.93
Low income (0.0 – 1470.0)	1.16	0.96	1.40	0.12
Diabetes, yes	0.95	0.79	1.14	0.58
Birth country, Sweden	0.94	0.79	1.13	0.53
Transient ischemic attack, yes	0.51	0.38	0.68	< 0.001
Living in nursing homes	2.01	1.67	2.41	< 0.001
Living alone, yes	1.28	1.10	1.49	< 0.01

Abbreviations: OR, Odds Ratio; PM_{10}: the concentration of a particulate matter with a diameter of <10 micrometres in µg/m3 the year before the stroke. OR is for a 5-unit increment on PM_{10}. Outcome: moderate to severe stroke defined as ≥ 6 points on the National Institutes of health stroke scale (ref. mild stroke, ≤ 5). Income in 1000 SEK.

Statistics: Binary logistic regression, multivariable model, n = 3875.

eTable 4. Results of multivariable logistic regression analyses examining the associations between ischemic stroke and Equivalent Continuous Sound Level ($L_{Aeq,24h}$) one year before the index stroke.

Variable	OR	95 % Confidence Interval		P-value
$L_{Aeq,24h}$ (dB)	1.02	0.95	1.09	0.56
Female sex	0.96	0.77	1.18	0.70
Age	1.01	1.00	1.01	0.13
Atrial fibrillation, yes	1.17	0.93	1.50	0.19
University education (≥ 13 years), Ref				
Secondary school (10–12 years)	0.84	0.64	1.10	0.21
Primary school (≤9 years)	0.76	0.57	1.01	0.07
High income (2003.0 – 94009.0), Ref				
Medium income (1471.0 – 1999.0)	0.88	0.67	1.16	0.37
Low income (0.0 – 1470.0)	0.90	0.68	1.18	0.44
Diabetes, yes	1.43	1.08	1.92	0.01
Birth country, Sweden	1.05	0.80	1.35	0.73
Transient ischemic attack, yes	1.23	0.81	1.95	0.35
Living in nursing homes	0.93	0.70	1.23	0.59
Living alone, yes	0.92	0.74	1.15	0.46

Abbreviations: OR, odds ratio; $L_{Aeq,24h}$ an Equivalent Continuous Sound Level in dB, the year before the stroke. OR is for a 5-unit increment on $L_{Aeq,24h}$. Outcome: Ischemic stroke (ref., category Hemorrhagic stroke). Income in 1000 SEK.

Statistics: Binary logistic regression, multivariable model, n = 3875.

eTable 5. Results of multivariable logistic regression analyses examining the associations between ischemic stroke at admission and the concentration of Nitrogen Oxides (NOx) one year before index stroke.

Variable	OR	95 % Confidence interval		P-value
NOx (µg/m³)	1.01	0.95	1.08	0.75
Female sex	0.96	0.77	1.18	0.69
Age	1.01	1.00	1.01	0.13
Atrial fibrillation, yes	1.17	0.93	1.50	0.19
University education (≥ 13 years), Ref				
Secondary school (10–12 years)	0.84	0.64	1.10	0.21
Primary school (≤9 years)	0.76	0.57	1.01	0.06
High income (2003.0 – 94009.0), Ref				
Medium income (1471.0 – 1999.0)	0.88	0.67	1.16	0.36
Low income (0.0 – 1470.0)	0.90	0.68	1.18	0.44
Diabetes, yes	1.43	1.08	1.92	0.02
Birth country, Sweden	1.05	0.80	1.35	0.73
Transient ischemic attack, yes	1.23	0.81	1.95	0.35
Living in nursing homes	0.92	0.70	1.22	0.58
Living alone, yes	0.92	0.74	1.15	0.46

Abbreviations: OR, Odds Ratio; NOx: the concentration of Nitrogen Oxides in µg/m3 the year before the stroke. OR is for a 5-unit increment on NOx. Outcome: Outcome: Ischemic stroke (ref. category Hemorrhagic stroke). Income in 1000 SEK.

Statistics: Binary logistic regression, multivariable model, n = 3875.

eTable 6. Results of multivariable logistic regression analyses examining the associations between ischemic stroke and the concentration of a particulate matter with a diameter of <10 micrometres (PM_{10}) one year before index stroke.

Variable	OR	95 % Confidence Interval		P-value
PM_{10} (µg/m³)	1.09	0.85	1.42	0.49
Female sex	0.96	0.78	1.19	0.71
Age	1.01	1.00	1.01	0.13
Atrial fibrillation, yes	1.18	0.93	1.50	0.18
University education (≥ 13 years), Ref				
Secondary school (10–12 years)	0.84	0.64	1.10	0.21
Primary school (≤9 years)	0.76	0.57	1.01	0.07
High income (2003.0 – 94009.0), Ref				
Medium income (1471.0 – 1999.0)	0.88	0.67	1.16	0.37
Low income (0.0 – 1470.0)	0.89	0.68	1.18	0.43
Diabetes, yes	1.43	1.08	1.92	0.02
Birth country, Sweden	1.05	0.80	1.35	0.73
Transient ischemic attack, yes	1.23	0.81	1.95	0.34
Living in nursing homes	0.92	0.70	1.22	0.58
Living alone, yes	0.92	0.78	1.17	0.45

Abbreviations: OR, Odds Ratio; PM_{10}: the concentration of a particulate matter with a diameter of <10 micrometres in µg/m3 the year before the stroke. OR is for a 5-unit increment on PM_{10}. Outcome: Ischemic stroke (ref. category Hemorrhagic stroke). Income in 1000 SEK.

Statistics: Binary logistic regression, multivariable model, n = 3875.

eTable 7. Results of multivariable logistic regression analyses examining the associations between moderate to severe stroke at admission and 10 years mean of the concentration of a particulate matter with a diameter of <10 micrometres (PM_{10}).

Variable	OR	95 % Confidence Interval		P-value
PM_{10} ($\mu g/m^3$), 10-year mean	0.82	0.67	0.99	0.04
Female sex	1.31	1.13	1.52	< 0.001
Age	1.01	1.01	1.02	< 0.001
Atrial fibrillation, yes	2.05	1.76	2.39	< 0.001
University education (≥ 13 years), Ref				
Secondary school (10–12 years)	1.43	1.19	1.72	< 0.001
Primary school (≤9 years)	1.44	1.19	1.76	< 0.001
High income (2003.0 – 94009.0), Ref				
Medium income (1471.0 – 1999.0)	0.99	0.83	1.20	0.96
Low income (0.0 – 1470.0)	1.17	0.97	1.41	0.11
Diabetes, yes	0.95	0.79	1.14	0.58
Birth country, Sweden	0.94	0.79	1.13	0.54
Transient ischemic attack, yes	0.51	0.38	0.68	< 0.001
Living in nursing homes	2.00	1.67	2.41	< 0.001
Living alone, yes	1.28	1.10	1.50	< 0.01

Abbreviations: OR, Odds Ratio; PM_{10}: the concentration of a particulate matter with a diameter of <10 micrometres in µg/m3. OR is for a 5-unit increment on PM_{10}. Outcome: moderate to severe stroke defined as ≥ 6 points on the National Institutes of health stroke scale (ref. category mild stroke, ≤ 5). Income in 1000 SEK.

Statistics: Binary logistic regression, multivariable model, n = 3875.

eTable 8. Results of multivariable logistic regression analyses examining the associations between moderate to severe stroke at admission and 10 years mean of Equivalent Continuous Sound Level ($L_{Aeq,24h}$).

Variable	OR	95 % Confidence Interval		P-value
$L_{Aeq,24h}$ (dB), 10-year mean	0.96	0.91	1.01	0.11
Female sex	1.31	1.13	1.52	< 0.001
Age	1.01	1.01	1.02	< 0.001
Atrial fibrillation, yes	2.06	1.76	2.40	< 0.001
University education (≥ 13 years), Ref				
Secondary school (10–12 years)	1.43	1.19	1.72	< 0.001
Primary school (≤9 years)	1.45	1.19	1.76	< 0.001
High income (2003.0 – 94009.0), Ref				
Medium income (1471.0 – 1999.0)	0.99	0.82	1.19	0.92
Low income (0.0 – 1470.0)	1.15	0.95	1.39	0.14
Diabetes, yes	0.94	0.79	1.13	0.56
Birth country, Sweden	0.94	0.78	1.13	0.52
Transient ischemic attack, yes	0.51	0.38	0.68	< 0.001
Living in nursing homes	2.00	1.67	2.41	< 0.001
Living alone, yes	1.28	1.10	1.50	< 0.01

Abbreviations: OR, odds ratio; $L_{Aeq,24h}$ an Equivalent Continuous Sound Level in dB. OR is for a 5-unit increment on $L_{Aeq,24h}$. Outcome: moderate to severe stroke defined as ≥ 6 points on the National Institutes of health stroke scale (ref. category mild stroke, ≤ 5). Income in 1000 SEK.

Statistics: Binary logistic regression, multivariable model, n = 3875

eTable 9. Results of multivariable logistic regression analyses examining the associations between moderate to severe stroke at admission and 10 years mean of the concentration of Nitrogen Oxides (NOx)

Variable	OR	95 % confidence interval		P-value
NOx (µg/m³), 10-year mean	0.97	0.92	1.01	0.12
Female sex	1.31	1.13	1.52	< 0.001
Age	1.01	1.01	1.02	< 0.001
Atrial fibrillation, yes	2.06	1.76	2.40	< 0.001
University education (≥ 13 years), Ref				
Secondary school (10–12 years)	1.43	1.19	1.72	< 0.001
Primary school (≤9 years)	1.44	1.19	1.76	< 0.001
High income (2003.0 – 94009.0), Ref				
Medium income (1471.0 – 1999.0)	0.99	0.83	1.20	0.96
Low income (0.0 – 1470.0)	1.16	0.96	1.40	0.12
Diabetes, yes	0.95	0.79	1.14	0.57
Birth country, Sweden	0.94	0.79	1.13	0.54
Transient ischemic attack, yes	0.51	0.38	0.68	< 0.001
Living in nursing homes	2.00	1.67	2.41	< 0.001
Living alone, yes	1.28	1.10	1.50	< 0.01

Abbreviations: OR, Odds Ratio; NOx: the concentration of Nitrogen Oxides in µg/m3. OR is for a 5-unit increment on NOx. Outcome: moderate to severe stroke defined as ≥ 6 points on the National Institutes of health stroke scale (ref. category mild stroke, ≤ 5). Income in 1000 SEK.

Statistics: Binary logistic regression, multivariable model, n = 3875.

eTable 10. Results of multivariable logistic regression analyses examining the associations between ischemic stroke and a 10-year mean of the Equivalent Continuous Sound Level ($L_{Aeq,24h}$).

Variable	OR	95 % Confidence Interval		P-value
$L_{Aeq,24h}$ (dB), 10-year mean	1.05	0.97	1.12	0.20
Female sex	0.96	0.78	1.19	0.70
Age	1.01	1.00	1.01	0.14
Atrial fibrillation, yes	1.18	0.93	1.50	0.18
University education (≥ 13 years), Ref				
Secondary school (10–12 years)	0.84	0.64	1.10	0.23
Primary school (≤9 years)	0.76	0.57	1.07	0.07
High income (2003.0 – 94009.0), Ref				
Medium income (1471.0 – 1999.0)	0.88	0.67	1.16	0.37
Low income (0.0 – 1470.0)	0.90	0.68	1.18	0.45
Diabetes, yes	1.43	1.08	1.93	0.01
Birth country, Sweden	1.05	0.80	1.36	0.73
Transient ischemic attack, yes	1.24	0.82	1.96	0.34
Living in nursing homes	0.93	0.703	1.23	0.59
Living alone, yes	0.92	0.74	1.14	0.45

Abbreviations: OR, odds ratio; $L_{Aeq,24h}$ an Equivalent Continuous Sound Level in dB. OR is for a 5-unit increment on $L_{Aeq,24h}$. Outcome: Ischemic stroke (ref. category Hemorrhagic stroke). Income in 1000 SEK.

Statistics: Binary logistic regression, multivariable model, n = 3875.

eTable 11. Results of multivariable logistic regression analyses examining the associations between ischemic stroke and a 10-year mean of the concentration of Nitrogen Oxides (NOx).

Variable	OR	95 % Confidence Interval		P-value
NOx (µg/m³), 10-year mean	1.01	0.95	1.08	0.70
Female sex	0.96	0.77	1.18	0.69
Age	1.01	1.00	1.01	0.13
Atrial fibrillation, yes	1.18	0.93	1.50	0.18
University education (≥ 13 years), Ref				
Secondary school (10–12 years)	0.84	0.64	1.10	0.21
Primary school (≤9 years)	0.76	0.57	1.01	0.06
High income (2003.0 – 94009.0), Ref				
Medium income (1471.0 – 1999.0)	0.88	0.67	1.15	0.36
Low income (0.0 – 1470.0)	0.90	0.68	1.18	0.43
Diabetes, yes	1.43	1.08	1.92	0.02
Birth country, Sweden	1.05	0.80	1.35	0.73
Transient ischemic attack, yes	1.23	0.81	1.95	0.35
Living in nursing homes	0.92	0.70	1.22	0.58
Living alone, yes	0.92	0.74	1.15	0.46

Abbreviations: OR, Odds Ratio; NOx: the concentration of Nitrogen Oxides in µg/m3. OR is for a 5-unit increment on NOx. Outcome: Outcome: Ischemic stroke (ref. category Hemorrhagic stroke). Income in 1000 SEK.

Statistics: Binary logistic regression, multivariable model, n = 3875.

eTable 12. Results of multivariable logistic regression analyses examining the associations between ischemic stroke and a 10-year mean of the concentration of a particulate matter with a diameter of <10 micrometres (PM_{10}).

Variable	OR	95 % Confidence Interval		P-value
PM_{10} (µg/m³), 10-year mean	1.16	0.87	1.54	0.31
Female sex	0.96	0.78	1.19	0.70
Age	1.01	1.00	1.01	0.14
Atrial fibrillation, yes	1.18	0.93	1.51	0.18
University education (≥ 13 years), Ref				
Secondary school (10–12 years)	0.84	0.64	1.10	0.22
Primary school (≤9 years)	0.76	0.57	1.02	0.07
High income (2003.0 – 94009.0), Ref				
Medium income (1471.0 – 1999.0)	0.88	0.67	1.15	0.36
Low income (0.0 – 1470.0)	0.89	0.68	1.17	0.41
Diabetes, yes	1.43	1.08	1.92	0.02
Birth country, Sweden	1.05	0.80	1.35	0.74
Transient ischemic attack, yes	1.23	0.81	1.96	0.34
Living in nursing homes	0.93	0.70	1.23	0.59
Living alone, yes	0.92	0.74	1.15	0.46

Abbreviations: OR, Odds Ratio; PM_{10}: the concentration of a particulate matter with a diameter of <10 micrometres in µg/m3. OR is for a 5-unit increment on PM_{10}. Outcome: Ischemic stroke (ref. category Hemorrhagic stroke). Income in 1000 SEK.

Statistics: Binary logistic regression, multivariable model, n = 3875.
